# One Year of the COVID-19 Pandemic in Dental Medical Facilities in Germany: A Questionnaire-Based Analysis

**DOI:** 10.3390/ijerph19010175

**Published:** 2021-12-24

**Authors:** Stephan Zellmer, Ella Bachmann, Anna Muzalyova, Alanna Ebigbo, Maria Kahn, Claudia Traidl-Hoffmann, Roland Frankenberger, Fabian M. Eckstein, Thomas Ziebart, Axel Meisgeier, Helmut Messmann, Christoph Römmele, Tilo Schlittenbauer

**Affiliations:** 1 Department of Internal Medicine III—Gastroenterology and Infectious Diseases, University Hospital Augsburg, Stenglinstraße 2, 86156 Augsburg, Germany; anna.muzalyova@uk-augsburg.de (A.M.); alanna.ebigbo@uk-augsburg.de (A.E.); maria_kahn@gmx.de (M.K.); Helmut.Messmann@uk-augsburg.de (H.M.); Christoph.Roemmele@uk-augsburg.de (C.R.); 2 Department of Oral and Maxillofacial Surgery, University Hospital Augsburg, Sauerbruchstraße 6, 86179 Augsburg, Germany; Tilo.Schlittenbauer@uk-augsburg.de; 3 Department of Environmental Medicine, Faculty of Medicine, University of Augsburg, Neusäßer Straße 47, 86156 Augsburg, Germany; Claudia.Traidl-Hoffmann@uk-augsburg.de; 4 Department of Operative Dentistry, Endodontics and Pediatric Dentistry, Campus Marburg, University Medical Center Giessen and Marburg, Georg-Voigt-Str. 3, 35039 Marburg, Germany; frankbg@med.uni-marburg.de; 5 Department of Oral and Maxillofacial Surgery, Hannover Medical School, 30625 Hannover, Germany; Eckstein.Fabian@mh-hannover.de; 6 Department of Oral and Maxillofacial Surgery, Campus Marburg, Philipps University Marburg and University Medical Center Giessen and Marburg, Baldingerstr, 35033 Marburg, Germany; ziebart@med.uni-marburg.de (T.Z.); mkg@med.uni-marburg.de (A.M.)

**Keywords:** COVID-19, dentistry, healthcare

## Abstract

(1) Background: The COVID-19 pandemic forced healthcare workers to adapt to challenges in both patient care and self-protection. Dental practitioners were confronted with a potentially high possibility of infection transmission due to aerosol-generating procedures. This study aims to present data on healthcare worker (HCW) screening, infection status of HCWs, pre-interventional testing, the use of personal protective equipment (PPE) and the economic impact of the pandemic in dental facilities. (2) Methods: Dental facilities were surveyed nationwide using an online questionnaire. The acquisition of participants took place in cooperation with the German Society for Dentistry, Oral and Maxillofacial Medicine. (3) Results: A total of 1094 private practices participated. Of these, 39.1% treated fewer than 600 patients per quarter and 59.9% treated over 600 patients per quarter. Pre-interventional testing was rarely performed in either small (6.6%) or large practices (6.0%). Large practices had a significantly higher incidence of at least one SARS-CoV-2-positive HCW than small practices (26.2% vs.14.4%, *p* < 0.01). The main source of infection in small practices was the private environment, and this was even more significant in large practices (81.8% vs. 89.7%, *p* < 0.01). The procedure count either remained stable (34.0% of small practices vs. 46.2% of large practices) or decreased by up to 50% (52.6% of small practices vs. 44.4% of large practices). Revenue remained stable (24.8% of small practices vs. 34.2% of large practices) or decreased by up to 50% (64.5% of small practices vs. 55.3% of large practices, *p* = 0.03). Overall, employee numbers remained stable (75.5% of small practices vs. 76.8% of large practices). A vaccination readiness of 60–100% was shown in 60.5% (*n* = 405) of large practices and 59.9% (*n* = 251) of small practices. (4) Conclusion: Pre-interventional testing in dental practices should be increased further. Economic challenges affected small practices as well as large practices. Overall, a steady employee count could be maintained. Vaccination readiness is high in dental practices, although with some room for improvement.

## 1. Introduction

Initially reported in Wuhan, China, at the end of 2019, the novel severe acute respiratory syndrome coronavirus type 2 (SARS-CoV-2) has spread rapidly, resulting in the World Health Organization officially declaring COVID-19 a pandemic on March 11 2020 [1]. Since the beginning of the COVID-19 pandemic, Germany has experienced multiple waves of infection [2].

To lower infection rates, precautionary measures have been implemented in many parts of the world, including social distancing, hygiene measures (washing hands, sneeze/cough etiquette), face masks and room ventilation [3].

With millions of cases worldwide, the SARS-CoV-2 pandemic confronted healthcare workers with a multitude of challenges ranging from the care of infected patients and self-protection to economic challenges [4,5]. A redistribution of the work force and supplies was initiated, focusing mainly on intensive care units and COVID-19 hospital wards [6,7]. Personal protective equipment such as FFP2/3 masks were distributed in hospitals in the early phase of the pandemic. However, the situation was different in private practices, especially regarding dental health. Not partaking in COVID-19 patient care, dental health workers in Germany felt left out of the discussion regarding safety protocols and personal protective equipment, as well as economic compensation [8].

For dentists working in proximity to patients’ uncovered facial areas during interventions, the risk of SARS-CoV-2 transmission via aerosols and droplets seemed especially high [9]. As aerosol-generating procedures (AGP) are a routine part of dental medicine, a closer inspection of infection rates and pre-interventional testing in this medical discipline is required.

Many dentists were aware of the necessity of using appropriate protective equipment early in the pandemic [10]. However, the recommendations of various dental societies on the use of PPE are partially inconsistent. Therefore, a closer look at the use of PPE is important [11].

In collaboration with the B-FAST project of the NUM (Network of University Medicine) the University Hospital Augsburg surveyed data on medical fields with aerosol-generating procedures (AGP), such as gastroenterology, otolaryngology, oral and maxillofacial surgery and dentistry.

This study aims to present data from a nationwide survey of dental facilities regarding the COVID-19 pandemic. In particular, the use of PPE, pre-interventional testing, healthcare worker screening, healthcare worker status and pandemic-related economic changes at the facilities were examined.

## 2. Materials and Methods

Based on expert discussion and a review of the current literature, a self-report online questionnaire with 64 items was designed (Supplementary Material Figure S1). This questionnaire was sent to private practices and clinics all over Germany and was available from 16 December to 24 January. Specialties with aerosol-generating procedures, such as gastroenterology, otolaryngology, oral and maxillofacial surgery and dentistry were included (Table 1). Topics covering infection of healthcare workers, personal protective equipment, pre-interventional testing, development of procedures, revenue, employee numbers and vaccination readiness during the COVID-19 pandemic were included. All questions were distributed between April 2020 and December 2020.

This publication focuses only on the subcategory of dentistry and compares the consequences of the pandemic for small practices and large practices. A small practice was defined as a practice treating at most 600 patients per quarter, and practices above this threshold were assigned to the category of large practices. The present paper focuses on the analysis of data provided by private practices only, as clinics were not sufficiently represented in the study.

The nationwide study addressed heads of department and owners of dental facilities. Recruitment took place via the German Society for Dentistry and Oral Medicine (DGZMK). The online questionnaire could be answered from 16 December 2020 to 24 January 2021.

The statistical analysis for the dentistry subgroup was performed using IBM^®^ SPSS version 27.0 (IBM, New York City, NW, USA). Associations between categorical variables were assessed using the chi-squared test or Fisher’s exact test where appropriate. Comparison of related samples was performed using the Friedman test. The significance level was set at *p* < 0.05.

The study was conducted in accordance with the Declaration of Helsinki and the Good Clinical Practice (GCP) guidelines. A positive ethical evaluation of the study was obtained from the Ethics Committee of the Faculty of Medicine of the Technical University of Munich under the accession number 713/20 S-S.

## 3. Results

### 3.1. Sample

Overall, 1114 dental medical facilities filled out the online questionnaire. Among the participating dental medical facilities, the majority were private practices, whereas clinics constituted only 1.8% of the study population. Of the private practices, 38.2% (*n* = 426) treated fewer than 600 patients per quarter and 59.9% (*n* = 668) treated 600 patients or more per quarter (Table 1).

### 3.2. Healthcare Worker Status

The number of SARS-CoV-2-positive HCWs in private practices and hospital-based departments is shown in Table 2. Overall, 3.3% (*n* = 396) of HCWs had a SARS-CoV-2 infection, confirmed via PCR testing. A significantly higher number of HCWs tested positive for SARS-CoV-2 in private practices compared to hospitals (3.4%, *n* = 382 vs. 2.1%, *n* = 14, *p* < 0.01). There was no significantly relevant difference regarding the infection rate between small and large practices, although large practices reported a slightly higher proportion of infected HCWs (3.5% vs. 3.1%, *p* = 0.371).

### 3.3. Source of Infection

In the following section, the assumed source of infection among HCWs identified by the heads of the facilities (heads of departments in hospitals or private practice owners) is specified. Small dental practices had at least one SARS-CoV-2-positive case among HCWs less frequently than large practices (14.4%, *n* = 61 vs. 26.2%, *n* = 175, *p* < 0.01) (Table 3). In small practices (91.8%, *n* = 56) as well as large private practices (89.7%, *n* = 157), the main source of infection was the private environment. However, small practices reported the private environment to be the primary source of infection significantly more often than large practices (*p* < 0.01). In addition, 10.4% (*n* = 7) of small practices reported an unclear origin of transmission, and 10.4% (*n* = 7) named the workplace without patient contact as the source of infection. In 12.6% (*n* = 22) of cases, large practices could not identify the source of infection.

### 3.4. Pre-Interventional Testing

It was reported that 93.4% (*n* = 397) of small practices and 94.0 % (*n* = 629) of large practices performed no pre-interventional testing (Table 4). If testing occurred, small practices (*n* = 20, 4.7%) as well as large practices (*n* = 25, 3.7%) most often used internal (carried out by the practices themselves) antigen testing (Table 4).

### 3.5. Personal Protective Equipment

**Figure 1:**
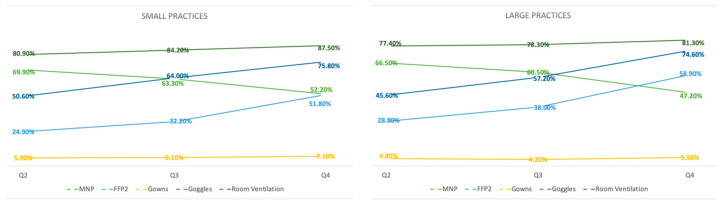
Personal protective equipment: usage from Q2 to Q4.

FFP2/3 use rose over the course of time in small practices (24.9% in Q2 to 52.8% in Q4, *p* < 0.01) as well as in large practices (28.8% in Q2 to 58.9% in Q4, *p* < 0.01), while MNP use (medical mouth–nose protection) declined in both small (69.9% in Q2 to 52.8% in Q4, *p* < 0.01) and large practices (66.5% in Q2 to 47.2% in Q4, *p* < 0.01). An increase was also observed in room ventilation in small practices (50.6% in Q2 to 75.8% in Q4, *p* < 0.01) and in large practices (45.6% in Q2 to 74.6% in Q4, *p* < 0.01). The use of gowns remained low in small (5.9% in Q2 to 7.1% in Q4, *p* = 0.194) and large practices (4.8% in Q2 to 5.5% in Q4, *p* = 0.275). The use of goggles remained at a constant level in small practices (80.9% in Q2 to 87.5% in Q4, *p* < 0.01) and in large practices (77.4% in Q2 to 81.3% in Q4, *p* < 0.01) (Figure 1).

### 3.6. Types of Procedures

The proportion of aerosol-generating procedures was less than 20% in 48.2% (*n* = 205) of small practices and 43.8% (*n* = 293) of large practices up to the end of 2020 (Table 5). Only 1.9% (*n* = 8) of small practices and 1.5% (*n* = 10) of large practices reported performing a proportion of aerosol-generating procedures of more than 80%.

The majority of small practices (*n* = 283, 66.6%) as well as the majority of large practices (*n* = 479, 71.6%) reported using a rubber dam in less than 20% of procedures.

### 3.7. Air Ventilation Systems

In large practices, ventilation systems were used more often than in small practices (50.9%, *n* = 339 vs. 43.7%, *n* = 185, *p* < 0.05) (Table 6). No significant difference could be identified regarding the use of different ventilation systems.

### 3.8. Development of Procedures

During the COVID-19 pandemic, the number of procedures performed remained stable in 34.0% (*n* = 144) of small practices and in 46.2% (*n* = 307) of large practices (Table 7). A decrease of less than 50% could be observed significantly more often in small private practices than in large private practices (52.6%, *n* = 223 vs. 44.4%, *n* = 295, *p* < 0.01). 

### 3.9. Economic Revenue and Employee Numbers

None of the large practices reported a revenue increase of over 50% (Table 7), whereas 0.7% (*n* = 3) of small private practices did so. At 8.8% (*n* = 59), a significantly higher number of large practices experienced an increase of less than 50% than small practices (3.8%, *n* = 16, *p* < 0.01). A stable revenue was reported significantly more often by large practices 34.2% (*n* = 228) than by small practices 24.8% (*n* = 105, *p* < 0.01). A decrease in revenue of less than 50% was observed in 55.3% (*n* = 369) of large practices and significantly more often in small practices, at 64.5% (*n* = 273, *p* < 0.01) (Table 8).

Employee numbers remained stable in 76.8% (*n* = 514) of large practices and 75.7% (*n* = 321) of small practices. A significant increase in the size of the workforce was reported by 10.2% (*n* = 68) of large and 6.1% (*n* = 26) of small practices (*p* < 0.05) compared to a significant decrease in numbers in 13.0% (*n* = 87) of large and 18.4% (*n* = 78) of small practices (*p* < 0.05) (Table 8).

### 3.10. Vaccination Readiness

Table 9 shows the vaccination readiness in small and large practices. Vaccination readiness in the range between 80 and 100% was reported significantly more often in small than in large practices (37.6%, *n* = 160 vs. 28.7%, *n* = 192, *p* < 0.01). Large practices reported vaccination readiness of 60–<80% significantly more often than small practices (31.8%, *n* = 213 vs. 21.4%, *n* = 9, *p* < 0.01). The remaining vaccination readiness rates of 40–<60%, 20–<40%, and <20% were distributed among large and small practices relatively equally.

## 4. Discussion

This study presents the results of a nationwide survey of dental facilities and reports on the data regarding pre-interventional testing, HCW infection rates, use of personal protective equipment, economic turnover, and staff development during the COVID-19 pandemic. A total of 1094 private practices and 20 dental clinics participated. To place this in context, there are about 87 dentists per 100,000 inhabitants in Germany; about 50,000 dental practices in total and an unknown number of dental clinics [12].

Considering the proximity of dental HCWs to the facial area of the patient during interventions and the utilization of instruments generating aerosols and droplets containing potentially infectious material, patient testing beforehand could be an effective measure to decrease the risk of infection transmission [9]. However, only a small number of practices reported any pre-interventional testing, thus demonstrating a deficit in testing strategies in everyday dental patient care. Within these limited testing routines for patients, internal antigen testing was the most common in all practices. However, antigen tests have been shown to provide results that are not as accurate as those of PCR tests [13].

Taking this into account, the German Society for Dentistry, Oral and Maxillofacial Medicine (DGZMK) developed an S1 guideline in September 2020 regarding patient care during the COVID-19 pandemic. This guideline included no recommendation for pre-interventional testing; however, it addressed the possible necessity of implementing testing protocols depending on regional incidence rates [14]. Nevertheless, screening strategies are a cornerstone of SARS-CoV-2 infection prevention [15].

SARS-CoV-2 infections among HCWs occurred more often in large practices than in small practices. In both small and large practices, the rate of SARS-CoV-2-positive HCWs was significantly higher than in dental clinics [16]. Possibly, insufficient pre-interventional testing in practices could have been responsible for this. The S1 guideline of the DGZMK stated a lack of data on healthcare worker screening, so no consensus has been found to date [13]. Although this study shows that the main source of infection seems to be private and home environments, any possibility of reducing the spread of infection should be seized. A significant upward trend in the use of FFP2/3 masks in all practices, as well as in room ventilation, was observed. Safety goggles, already being used in many dental offices, showed slightly increased use in all practices. A similar positive trend in the usage of FFP2/3 masks or goggles could also be identified in studies from Poland, Turkey and Italy [17,18].

The DGZMK guideline recommends wearing a standard MNP during all patient interventions, whereas further protective measures such as FFP2/3 masks are only required when treating suspected or confirmed SARS-CoV-2 cases. Consequently, SARS-CoV-2-positive patients should only be treated as emergency cases, preferably in dental clinics with more resources, and they should be separated from other patients to avoid cross-contamination [13].

In this study, large practices reported using air ventilation systems significantly more often than small practices, with no significant difference in the type of air ventilation system used. Although the use of ventilation systems initially had only a minor role in the recommendations of various dental societies for managing COVID-19, by the end of 2020 more than half of large practices reported using air ventilation systems [11,14,19]. Several studies have demonstrated the positive effect of ventilation systems on aerosol exposure in rooms in dental clinics and dental medical practices [20,21]. Even aspiration systems, which were not investigated in this study, may play a role in the reduction of aerosols in dental medical facilities [22]. Furthermore, it has been shown that the type of aspiration system used influences the prevalence of SARS-CoV-2 infections [23]. Therefore, attention should be given to using ventilation systems and adequate aspiration systems.

The economic effects of the COVID-19 pandemic on the dental health sector did not go unnoticed. A decline in revenue of up to 50% was reported in the majority of practices, while about a quarter of small practices and approximately one third of larger practices registered no economic change. An increase in revenue of up to 50% was reported in 3.8% of small and 8.8% of large practices. This increase in income contrasts with a simulation by Schwendicke et al. which predicted a substantial loss of revenue for both dental medical practices and dental clinics [24]. This increase could correlate with the overall decrease in revenue in dental practices. To some extent, larger practices may have been handling the situation differently compared with smaller practices [25]. With possibly more staff and financial resources to support pandemic-related measures, some larger practices may have been able to offer treatment while other dental practices had to shut down or decrease their patient flow, thereby taking on new patients and subsequently profiting indirectly. In addition, the guideline of the German Society of Dentistry recommended that suspected or confirmed COVID-19 cases should preferably be treated in specialized facilities, thereby benefitting individual practices [14]. Following the same trend, 52.6% of small practices and 44.4% of large practices suffered a decline in the number of procedures of up to 50%. A survey conducted in June 2020 at the waning of the first wave reported a reduction in workload of about 60% in both small and large dental facilities [25]. The difference with respect to the data of the present study, which were collected during the second wave of the pandemic, could be explained by a change in the way the pandemic was handled. An increase in procedures of up to 50% was seen in a small number of practices. Even given the fluctuations in revenue and procedure counts, most of the small and large practices were able to maintain a stable staff size. Compensation for loss of income was discussed in a press statement by the KZVB on 22 March 2021, regarding financial support for dentists up to a maximum of EUR 275,000,000 as from 1 April 2021 [26].

In this study, we were able to demonstrate a high vaccination readiness in most practices. However, no further inquiry was made containing interest in certain manufacturers or types of vaccines. While the initial hesitation towards the newly developed vaccines was widespread, vaccination rates increased steadily after their introduction and after vaccines became generally available for the population as a whole [27].

As in all cross-sectional studies, our study has several limitations. A selection bias cannot be eliminated due to the method of data collection via email from the German Society for Dentistry, Oral and Maxillofacial Medicine. Most importantly, various factors could influence the objectivity of the answers, as the participating head of department or private practice owner provided an assessment for the entire facility.

## 5. Conclusions

The rate of infection among HCWs was significantly higher in private practices than in hospital wards, and significantly higher in large practices than in small practices. Pre-interventional testing was implemented in only a few dental facilities, and therefore this requires improvement. Ventilation systems were used in approximately half of the dental practices, and their use should be expanded. Finally, the economic impact of the pandemic on dental facilities was evident; however, the number of employees in most facilities remained unchanged during the pandemic.

## Figures and Tables

**Table 1:** Distribution by patients per quarter in private practices.

		N	%
Total		1114	100
Hospital		20	1.8
Private Practice		1094	98.2
	Small (<600 ppq)	425	38.2
	Large (>600 ppq)	669	60.1

N: number of facilities; ppq: patients per quarter.

**Table 2:** Distribution of SARS-CoV-2-positive healthcare workers in questioned private practices.

	Employees (Total)		Employees (SARS-CoV-2-Positive)			
	N	HCW	N	HCW	Rate of SARS-CoV-2-Positive HCWs	*p*
Hospital	20	670	6	14	2.1	<0.01
Private Practice	1094	11,334	235	382	3.4	
Small (<600 ppq)	425	2534	61	78	3.1	n.s.
Large (>600 ppq)	669	8800	175	304	3.5	
Overall	1114	12,004	241	396	3.3	

N: number of facilities; HCW: healthcare worker; ppq: patients per quarter.

**Table 3:** Assumed source of infection for healthcare workers.

		Private Practices				
		Small (<600 ppq)		Large (>600 ppq)		
		N	%	N	%	p
Total		425	100.0	669	100.0	
Facilities with no SARS-CoV-2-positive HCW		364	85.6	494	73.8	
Facilities with SARS-CoV-2-positive HCWs		61	14.4	175	26.2	<0.01
	During interventions	0	0.0	3	1.7	n.s.
	At work (with patient contact)	0	0.0	4	2.3	n.s.
	At work (without patient contact)	7	11.5	7	4.0	n.s.
	Unclear origin	7	11.5	22	12.6	n.s.
	Private environment	56	91.8	157	89.7	<0.01

N: number of facilities; HCW: healthcare worker; ppq: patients per quarter. It was possible to select more than one source of infection.

**Table 4:** Methods and distribution of pre-interventional testing of outpatients and inpatients.

		Private Practices				
		Small (<600 ppq)		Large (>600 ppq)		
		N	%	N	%	*p*
Total		425	100	669	100	n.s.
	Internal PCR	0	0.0	4	0.6	n.s.
	Internal antigen	20	4.7	25	3.7	n.s.
	External PCR	6	1.4	5	0.7	n.s.
	External antigen	2	0.5	6	0.9	n.s.
	No testing	397	93.4	629	94.0	n.s.

N: number of facilities; PCR: polymerase chain reaction; ppq: patients per quarter.

**Table 5:** Share of aerosol-generating procedures and use of rubber dam.

		Private Practices				
	Share of Procedures	Small (<600 ppq)		Large (>600 ppq)		*p*-Value
	%	N	%	N	%	
Aerosol-generating procedures		425	100	669	100	
	<20	205	48.2	293	43.8	n.s.
	20–<40	152	35.8	254	38.0	n.s.
	40–<60	48	11.3	91	13.6	n.s.
	60–<80	12	2.8	21	3.1	n.s.
	>80	8	1.9	10	1.5	n.s.
Rubber dam		425	100	669	100	
	<20	283	66.6	479	71.6	n.s.
	20–<40	69	16.2	111	16.6	n.s.
	40–<60	36	8.5	47	7.0	n.s.
	60–<80	20	4.7	18	2.7	n.s.
	>80	17	4.0	14	2.1	n.s.

N: number of facilities; ppq: patients per quarter.

**Table 6:** Use of air ventilation systems.

		Private Practices				
		Small (<600 ppq)		Large (>600 ppq)		*p*-Value
		N	%	N	%	
Total		423	100	666	100	
Yes		185	43.7	339	50.9	<0.05
	Air handling units (AHU) in the recirculation mode	25	13.5	60	17.7	n.s.
	Air handling units (AHU) with air filter (e.g., HEPA filter) with/without recirculation mode	71	38.4	136	40.1	n.s.
	Fans/mobile air conditioners without air filter/fan heaters	53	28.6	78	23.0	n.s.
	Other types	24	13.0	38	11.2	n.s.
	Unknown	12	6.5	27	8.0	n.s.
No		237	56.0	319	47.9	<0.01
Unknown		1	0.2	8	1.2	n.s.

N: number of facilities; ppq: patients per quarter. Not all participants answered this question.

**Table 7:** Development of procedures.

		Private Practices				
		Small (<600 ppq)		Large (>600 ppq)		
		N	%	N	%	*p*
Total		424	100.0	664	100.0	
	Increase over 50%	10	2.4	11	1.7	n.s.
	Increase less than 50%	24	5.7	45	6.8	n.s.
	Stable	144	34.0	307	46.2	<0.01
	Decrease less than 50%	223	52.6	295	44.4	<0.01
	Decrease over 50%	23	5.4	6	0.9	<0.01

N: number of facilities; ppq: patients per quarter. Not all participants answered this question.

**Table 8:** Development of procedures.

		Private Practice				
		Small (<600 ppq)		Large (>600 ppq)		
		N	%	N	%	*p*
Revenue		423	100	667	100	
	Increase over 50%	3	0.7	0	0	n.s.
	Increase less than 50%	16	3.8	59	8.8	<0.01
	Stable	105	24.8	228	34.2	<0.01
	Decrease less than 50%	273	64.5	369	55.3	<0.05
	Decrease over 50%	26	6.1	11	1.6	<0.01
Employees		425	100	669	100	
	Increase	26	6.1	68	10.2	<0.05
	Stable	321	75.5	514	76.8	n.s.
	Decrease	78	18.4	87	13	<0.05

N: number of facilities; ppq: patients per quarter. Not all participants answered this question.

**Table 9:** Vaccination readiness by size of the private practice.

		Private Practice				
	Vaccination Readiness	Small (<600 ppq)		Large (>600 ppq)		
	%	N	%	N	%	*p*
Total		425	100	669	100	
	<20	27	6.4	40	6.0	n.s.
	20–<40	46	10.8	70	10.5	n.s.
	40–<60	101	23.8	154	23.0	n.s.
	60–<80	91	21.4	213	31.8	<0.01
	80–100	160	37.6	192	28.7	<0.01

N: number of facilities; ppq: patients per quarter.

## Data Availability

Data can be obtained from the corresponding author on request.

## Supplementary Materials

The following are available online at https://www.mdpi.com/article/10.3390/ijerph19010175/s1. Figure S1: Questionnaire.
