# Supplementary material for: One Year of the COVID-19 Pandemic in Dental Medical Facilities in Germany: A Questionnaire-Based Analysis
Source: Int J Environ Res Public Health. 2021 Dec 24;19(1):175. doi: 10.3390/ijerph19010175 (PMC8750787; doi:10.3390/ijerph19010175)
Supplement: Supplementary file 1 [file ijerph-19-00175-s001.zip › Figure S1 Questionnaire.pdf]

1. Basic data

1.1 Is the location of your private practice/department in Bavaria?

- Yes
- No

1.1 What are the first two digits of your postcode?

FIGURE FIELD

1.2 Please enter below the first two letters of your mother's maiden name and the first two letters of your father's first name. This code serves to better allocate the two-sided questionnaire to each other and does not allow any conclusions to be drawn about your person/private practice/department:

FOUR DIGITS

1.3 Type of facility:

- Clinic (continue with 1.4.1)
- Private practice (continue with 1.5.1)

1.4.1 What is the level of care classification of your hospital?

- Primary care
- Secondary care
- Tertiary care
- Not specified

1.4.2 How many beds does your hospital have?

- Under 100 beds
- 100 to < 200 beds
- 200 to < 500 beds
- 500 beds to < 800 beds
- Over 800 beds

1.5.1 How many notes do you have per quarter?

- Under 600
- 600 to < 1200
- 1800 to < 2400
- Over 2400

1.6 How many employees work in the respective department (e.g. endoscopy department/ entire private practice)?

FIGURE FIELD

1.7 Related to question 1.6: What is the full-time position equivalent (cumulative)?

FIGURE FIELD

1.8 Which specialization do you belong to?

- Gastroenterology/Endoscopy
- ORL
- Dentistry
- OMS
- Other: ___________

2. Employee status since the start of the pandemic

2.1 How many employees had already been diagnosed with COVID-19?

NUMBER FIELD

2.2 How many of them are most likely to have caught it in the following situations?

In the private environment	At work (during interventions)	At work (other patient contact, e.g. registration)	At work (without patient contact, e.g. during the break)	Unclear origin of infection
FIGURE FIELD	FIGURE FIELD	FIGURE FIELD	FIGURE FIELD	FIGURE FIELD

2.3 How many employees in total were already in quarantine?

FIGURE FIELD

2.4 How many employees were quarantined due to job-related exposure?

FIGURE FIELD

2.5 Have you had to cancel procedures due to pandemic-related staff shortages?

- Yes
- No

2.6 Have you screened yourself and/or your staff for COVID-19 (testing in the asymptomatic without specific cause)?

- Yes (continue with 2.7.1)
- No (continue with 2.8)

2.7.1 What was the primary method?

- Antibody test

- PCR
- Antigen test
- other

2.7.2 Have you detected positive employees as part of a screening program?
- Yes
- No

2.7.3 Did the screening have any consequences?
- Yes
- No

2.8 What do you estimate the vaccination readiness among your clinic/private practice staff?
- up to < 20 %
- 20 % to < 40
- 40 % to < 60
- 60 % to < 80
- 80% to 100%

3a. Procedures (Gastroenterology/Endoscopy specific)

3a.1 How many procedures do you perform on average per year?
FIGURE FIELD

3a.2 What percentage of these are endoscopic procedures in the upper gastrointestinal tract (gastroscopy, ERCP, endosonography, etc.) or bronchoscopies?
- up to < 20 %
- 20 % to < 40
- 40 % to < 60
- 60 % to < 80
- 80% to 100%

3a.3 What percentage of these are endoscopic procedures in the lower gastrointestinal tract (colonoscopy, sigmoidoscopy, etc.)?
- 0 to < 20 %
- 20 % to < 40
- 40 % to < 60
- 60 % to < 80
- 80% to 100%

3a.4 How many examinations did you perform on average per working day in your private practice/department before the pandemic started?
FIGURE FIELD

3a.5 How many treatment rooms does your department/private practice have?
FIGURE FIELD

3a.6 What is the average size of your treatment rooms?
- < 10 sqm
- 10 to < 15 sqm
- 15 to < 20 sqm
- 20 to < 25 sqm
- > 25 sqm

3a.7 How many employees are typically in the room for a procedure?
FIGURE FIELD

3a.8 Are ventilation systems (ventilation and air-conditioning systems, fans, mobile air-conditioning units without air filters, fan heaters, etc.) installed?
- Yes (continue with 3a.8.1)
- No (continue with 4.1)
- Unknown (continue with 4.1)

3a.8.1 If yes: Which ventilation systems are mainly installed?
- Air handling units (AHU) in recirculation mode
- Air handling unit (AHU) with air filter (e.g. HEPA filter) with / without recirculation mode
- Fans / mobile air conditioners without air filter / fan heaters
- Other: _______________
- Unknown

3b. Procedures (ORL specific)

3b.1 How many endoscopic procedures do you perform on average per year?
NUMBER FIELD

3b.2 What percentage of these are flexible endoscopies?

- up to < 20 %
- 20 % to < 40
- 40 % to < 60
- 60 % to < 80
- 80% to 100%

3b.3 What percentage of these are laryngeal endoscopies?

- 0 to < 20 %
- 20 % to < 40
- 40 % to < 60
- 60 % to < 80
- 80% to 100%

3b.4 How many percent of these are microlaryngoscopies under general anaesthesia?

-0 to < 20 %
-20 % to < 40
-40 % to < 60
-60 % to < 80
-80 % to 100%

3b.5 How many examinations did you perform on average per working day in your private practice/department before the pandemic started?
FIGURE FIELD

3b.6 How many treatment rooms does your department/private practice have?
FIGURE FIELD

3b.7 What is the average size of your treatment rooms?

- < 10 sqm
- 10 to < 15 sqm
- 15 to < 20 sqm
- 20 to < 25 sqm
- > 25 sqm

3b.8 How many employees are typically in the room for a procedure?
FIGURE FIELD

3b.9 Are ventilation systems (ventilation and air-conditioning systems, fans, mobile air-conditioning units without air filters, fan heaters, etc.) installed?

- Yes (continue with 3b.9.1)
- No (continue with 4.1)
- Unknown (continue with 4.1)

3b.9.1 If yes: Which ventilation systems are mainly installed?

- Air handling units (AHU) in recirculation mode
- Air handling unit (AHU) with air filter (e.g. HEPA filter) with / without recirculation mode
- Fans / mobile air conditioners without air filter / fan heaters
- Other: _______________
- Unknown

3c. Procedures (Dentistry specific)

3c.1 What is the specialization of your institution? (Multiple answers possible)

- Implantology
- Periodontology
- Prosthetic dentistry
- Pediatric dentistry
- Restorative dentistry
- Endodontics
- Orthodontics
- Other: _______________

3c.2 What is the percentage of aerosol-generating procedures (professional teeth cleaning, etc.) performed in your private practice/department?

- up to < 20 %
- 20 % to < 40
- 40 % to < 60
- 60 % to < 80
- 80% to 100%

3c.3 What is the percentage of non-aerosol-generating procedures performed in your private practice/department?

- 0 to < 20 %
- 20 % to < 40
- 40 % to < 60
- 60 % to < 80
- 80% to 100%

3c.4 What is the percentage of procedures performed using a rubber dam?
- 0 to < 20 %
- 20 % to < 40
- 40 % to < 60
- 60 % to < 80
- 80 % to 100%

3c.5 How many examinations did you perform on average per working day in your private practice/department before the pandemic started?
FIGURE FIELD

3c.6 How many treatment rooms does your department/private practice have?
FIGURE FIELD

3c.7 What is the average size of your treatment rooms?
- < 10 sqm
- 10 to < 15 sqm
- 15 to < 20 sqm
- 20 to < 25 sqm
- > 25 sqm

3c.8 How many employees are typically in the room for a procedure?
FIGURE FIELD

3c.9 Are ventilation systems (ventilation and air-conditioning systems, fans, mobile air-conditioning units without air filters, fan heaters, etc.) installed?
- Yes (continue with 3c.9.1)
- No (continue with 4.1)
- Unknown (continue with 4.1)

3c.9.1 If yes: Which ventilation systems are mainly installed?
- Air handling units (AHU) in recirculation mode
- Air handling unit (AHU) with air filter (e.g. HEPA filter) with / without recirculation mode
- Fans / mobile air conditioners without air filter / fan heaters
- Other: _______________
- Unknown

3d. Procedures (OMS specific)

3d.1 What is the specialization of your institution? (Multiple answers possible)
- Implantology
- Orthognathic surgery / dysgnathia surgery
- Traumatology
- Tumor surgery
- Other: _______________

3d.2 What is the percentage of tooth extractions performed under intubation anaesthesia?
- up to < 20 %
- 20 % to < 40
- 40 % to < 60
- 60 % to < 80
- 80% to 100%

3d.3 What is the percentage of tooth extractions performed under local anaesthesia?
- up to < 20 %
- 20 % to < 40
- 40 % to < 60
- 60 % to < 80
- 80% to 100%

3d.4 What is the percentage of procedures performed extraorally?
- up to < 20 %
- 20 % to < 40
- 40 % to < 60
- 60 % to < 80
- 80% to 100%

3d.5 What is the percentage of procedures performed intraorally?
- up to < 20 %

- 20 % to < 40
- 40 % to < 60
- 60 % to < 80
- 80% to 100%

3d.6 What is the percentage of procedures performed in the following locations?

In the operating room?
- up to < 20 %
- 20 % to < 40
- 40 % to < 60
- 60 % to < 80
- 80% to 100%

In the intervention room?
- up to < 20 %
- 20 % to < 40
- 40 % to < 60
- 60 % to < 80
- 80% to 100%

In the dental chair?
- up to < 20 %
- 20 % to < 40
- 40 % to < 60
- 60 % to < 80
- 80% to 100%

3d.7 What is the percentage of aerosol-generating procedures performed in your private practice/department?
- up to < 20 %
- 20 % to < 40
- 40 % to < 60
- 60 % to < 80
- 80% to 100%

3d. 8 What is the percentage of non-aerosol-generating procedures performed in your private practice/department?
- 0 to < 20 %
- 20 % to < 40
- 40 % to < 60
- 60 % to < 80
- 80% to 100%

3d.9 What is the percentage of procedures performed using a rubber dam?
- 0 to < 20 %
- 20 % to < 40
- 40 % to < 60
- 60 % to < 80
- 80% to 100%

3d.10 How many examinations did you perform on average per working day in your private practice/department before the pandemic started?
FIGURE FIELD

3d.11 How many treatment rooms does your department/private practice have?
FIGURE FIELD

3d.12 What is the average size of your treatment rooms?
- < 10 sqm
- 10 to < 15 sqm
- 15 to < 20 sqm
- 20 to < 25 sqm
- > 25 sqm

3d.13 How many employees are typically in the room for a procedure?
FIGURE FIELD

3d.14 Are ventilation systems (ventilation and air-conditioning systems, fans, mobile air-conditioning units without air filters, fan heaters, etc.) installed?
- Yes (continue with 3d.14.1)
- No (continue with 4.1)
- Unknown (continue with 4.1)

3d.14.1 If yes: Which ventilation systems are mainly installed?
- Air handling units (AHU) in recirculation mode
- Air handling unit (AHU) with air filter (e.g. HEPA filter) with / without recirculation mode
- Fans / mobile air conditioners without air filter / fan heaters
- Other: _______________
- Unknown

4. Protective measures in 2nd, 3rd and 4th quarter of 2020

4.1 Have you changed your prophylactic protection measures because of the pandemic?
- Yes
- No

4.2 Have you and your employees used/are you and your employees using mouth and nose protection?

In the 2nd quarter of 2020 (04.-06.2020)
- Always
- Very frequently

In the 3rd quarter of 2020 (07.-09.2020)
- Always
- Very frequently

In the 4th quarter of 2020 (10.-12.2020)
- Always
- Very frequently

- Occasionally
- Rarely
- Never

- Occasionally
- Rarely
- Never

- Occasionally
- Rarely
- Never

4.3 Have you and your employees used/are you and your employees using FFP2/N95 or FFP3 mask?

In the 2nd quarter of 2020 (04.-06.2020)
- Always
- Very frequently
- Occasionally
- Rarely
- Never

In the 3rd quarter of 2020 (07.-09.2020)
- Always
- Very frequently
- Occasionally
- Rarely
- Never

In the 4th quarter of 2020 (10.-12.2020)
- Always
- Very frequently
- Occasionally
- Rarely
- Never

4.4 Have you and your employees used/are you and your employees using water-repellent protective gowns?

In the 2nd quarter of 2020 (04.-06.2020)
- Always
- Very frequently
- Occasionally
- Rarely
- Never

In the 3rd quarter of 2020 (07.-09.2020)
- Always
- Very frequently
- Occasionally
- Rarely
- Never

In the 4th quarter of 2020 (10.-12.2020)
- Always
- Very frequently
- Occasionally
- Rarely
- Never

4.5 Have you and your employees used/are you and your employees using glasses or protective goggles/visors?

In the 2nd quarter of 2020 (04.-06.2020)
- Always
- Very frequently
- Occasionally
- Rarely
- Never

In the 3rd quarter of 2020 (07.-09.2020)
- Always
- Very frequently
- Occasionally
- Rarely
- Never

In the 4th quarter of 2020 (10.-12.2020)
- Always
- Very frequently
- Occasionally
- Rarely
- Never

4.6 Did/Do you and your staff air the treatment room(s) between procedures?

In the 2nd quarter of 2020 (04.-06.2020)
- Always
- Very frequently
- Occasionally
- Rarely
- Never

In the 3rd quarter of 2020 (07.-09.2020)
- Always
- Very frequently
- Occasionally
- Rarely
- Never

In the 4th quarter of 2020 (10.-12.2020)
- Always
- Very frequently
- Occasionally
- Rarely
- Never

4.7 Are you likely to change the prophylactic protective measures of your employees after approval of a vaccine against COVID-19?
- Yes
- No
- Unknown

5. Pre-interventional testing

5.1 Did/Do you pre-interventionally test your patients for COVID-19 or request/demand a current (externally performed) test? (Multiple answers possible)
- We tested/test pre-interventionally for SARS-CoV2 (continue with 5.1.1)
- We requested/request an actual test for SARS-CoV2 (continue with 5.1.2)
- We used/are using a different testing procedure for SARS-CoV2.
- We did not/do not perform pre-interventional testing for SARS-CoV2.

5.1.1 How long prior to the intervention was/is testing for COVID-19 mainly performed?

In the 2nd quarter of 2020 (04.-06.2020)
- < 24h
- 24 -< 48h
- 48h -< 72h
- >72h

In the 3rd quarter of 2020 (07.-09.2020)
- < 24h
- 24 -< 48h
- 48h -< 72h
- >72h

In the 4th quarter of 2020 (10.-12.2020)
- < 24h
- 24 -< 48h
- 48h -< 72h
- >72h

5.1.2 In which time period prior to the procedure did/do the test have to take place?

In the 2nd quarter of 2020 (04.-06.2020)
- 1 day (24h)
- 2 days (48h)
- 3 days (72h)
- 4 days (96h)
- Older

In the 3rd quarter of 2020 (07.-09.2020)
- 1 day (24h)
- 2 days (48h)
- 3 days (72h)
- 4 days (96h)
- Older

In the 4th quarter of 2020 (10.-12.2020)
- 1 day (24h)
- 2 days (48h)
- 3 days (72h)
- 4 days (96h)
- Older

5.2 Have you examined/treated suspected or known Covid-19 positive patients?

- Yes (continue with 5.3.1)
- No (continue with 6.1)

5.3.1 What is the number of examined/treated suspected cases?

FIGURE FIELD

5.3.2 What is the number of examined/treated COVID-19 positive patients?

FIGURE FIELD

6. Economic situation

6.1 What is the expected change in the number of procedures in 2020 compared to the previous year?

- Increase by more than 50%
- Increase by less than 50%
- Roughly constant
- Decrease by less than 50%
- Decrease by more than 50%

6.2 What is your estimated turnover development in 2020 compared to the previous year?

- Increase by more than 50%
- Increase by less than 50%
- Roughly constant
- Decrease by less than 50%
- Decrease by more than 50%

6.3 What is the trend in your employee number since the beginning of the pandemic (cumulative number of full-time positions)?

- Increase
- Roughly constant
- Decrease

6.4 Was this trend directly caused by the pandemic?

- Strongly agree
- Agree
- Disagree
- Strongly disagree

6.5 Have you postponed/are you postponing procedures because of the pandemic?

In the 2nd quarter of 2020 (04.-06.2020)

- Never
- Cancellation of up to 50% of elective examinations
- Cancellation of more than 50% of elective examinations
- Cancellation of all elective examinations (continue with 6.5.1)

In the 3rd quarter of 2020 (07.-09.2020)

- Never
- Cancellation of up to 50% of elective examinations
- Cancellation of more than 50% of elective examinations
- Cancellation of all elective examinations (continue with 6.5.1)

In the 4th quarter of 2020 (10.-12.2020)

- Never
- Cancellation of up to 50% of elective examinations
- Cancellation of more than 50% of elective examinations
- Cancellation of all elective examinations (continue with 6.5.1)

6.5.1 What were/are the main causes for the postponement? (Multiple answers possible)

In the 2nd quarter of 2020 (04.-06.2020)

- Voluntary preventive
- Ordered by public health department
- Staff shortage
- Patient shortage

In the 3rd quarter of 2020 (07.-09.2020)

- Voluntary preventive
- Ordered by
- Health Department
- Staff shortage
- Patient shortage

In the 4th quarter of 2020 (10.-12.2020)

- Voluntary preventive
- Ordered by public health department
- Staff shortage
- Patient shortage

Additionally for private practices:

6.6 Do you have loss of earnings insurance?

- Yes
- No
- Unknown

7. Serological study

If 1.1 is answered with "Yes":

7.1 Do you want to participate in our antibody test study?

- Yes
- No
